# Reliability and Validity of the Variability Model Testing Procedure for Somatic Dysfunction Assessment: A Comparison with Gait Analysis Parameters in Healthy Subjects

**DOI:** 10.3390/healthcare12020175

**Published:** 2024-01-11

**Authors:** Luca Vismara, Andrea Bergna, Andrea Gianmaria Tarantino, Fulvio Dal Farra, Francesca Buffone, Davide Vendramin, Veronica Cimolin, Serena Cerfoglio, Luca Guglielmo Pradotto, Alessandro Mauro

**Affiliations:** 1 Division of Neurology and Neurorehabilitation—IRCCS Istituto Auxologico Italiano, Strada Luigi Cadorna 90, 28824 Piancavallo-Verbania, Italy; lucavisma@hotmail.com (L.V.); veronica.cimolin@polimi.it (V.C.); serena.cerfoglio@polimi.it (S.C.); lucaguglielmo.pradotto@unito.it (L.G.P.); alessandro.mauro@unito.it (A.M.); 2 Department of Research, SOMA Istituto Osteopatia Milano—Institute Osteopathy Milan, 20126 Milan, Italy; andreabergna@soma-osteopatia.it (A.B.); andreagtarantino@gmail.com (A.G.T.); fulviodalfarra@outlook.it (F.D.F.); 3 Division of Paediatric, Manima Non-Profit Organization Social Assistance and Healthcare, 20125 Milan, Italy; davvendra@gmail.com; 4 Department of Information Engineering, University of Brescia, 25123 Brescia, Italy; 5 Principles and Practice of Clinical Research (PPCR), Harvard T.H. Chan School of Public Health–ECPE, Boston, MA 02115, USA; 6 Department of Electronics, Information and Bioengineering, Politecnico di Milano, Piazza Leonardo da Vinci 32, 20133 Milan, Italy; 7 Department of Neurosciences “Rita Levi Montalcini”, University of Turin, 10126 Turin, Italy

**Keywords:** somatic dysfunction, variability model, gait analysis, palpatory diagnosis, motion analysis

## Abstract

Somatic dysfunction (SD) is an altered body function involving the musculoskeletal system. However, its clinical signs—tissue texture abnormalities, positional asymmetry, restricted range of motion, and tissue tenderness—did not achieve satisfactory results for reliability. A recent theoretical model proposed a revision assessing the movement variability around the joint rest position. The asymmetry and restriction of motion may characterize functional assessment in osteopathic clinical practice, demonstrating the reliability required. Hence, this study investigated the reliability of the new variability model (VM) with gait analysis (GA). Three blind examiners tested 27 young healthy subjects for asymmetry of motion around rest position and the SD grade on six body regions. The results were compared to the VICON procedure for 3D-GA. The inter-rater agreement for the detection of reduced movement variability ranged from 0.78 to 0.54, whereas for SD, grade ranged from 0.64 to 0.47. VM had a sensitivity and specificity of 0.62 and 0.53, respectively, in SD detection compared to step length normality. Global severity grade of SD demonstrated moderate to good correlation with spatial-temporal parameters. The VM showed palpatory reliability and validity with spatial–temporal parameters in GA. Those findings contribute to the innovation for SD examination with implications for the clinical practice.

## 1. Introduction

Somatic dysfunction (SD) may influence the normal physiology of the body system, leading to potential health disorders [1,2,3]. SD is considered an “impaired or altered function of related components of the body framework system” [4], and the International Classification of Diseases defines it as a “biomechanical lesion not elsewhere classified”, proposing different body regions as possible locations [5]. However, even if it is widely used as a diagnostic target in both the clinical and research settings in osteopathy [6], there is an ongoing debate concerning the reliability and validity of the procedures to detect SD [7,8]. Furthermore, it has yet to be shown whether SD and health status are effectively related to each other [9]. According to the literature, osteopaths can identify the SD using four distinct clinical signs: tissue texture abnormalities, asymmetry, restricted range of motion (ROM), and tenderness (this procedure is commonly summarized by the TART acronym). However, the TART procedure did not reach satisfactory results in terms of reliability and validity [7], although training and consensus can moderately improve inter-operator agreement [10]. For this reason, TART clinical signs are considered the result of empirical observations that—taken individually—are scientifically questionable in the context of a palpatory assessment [7]. Only the “tenderness” parameter (i.e., the presence of a painful area evoked by the examiner manual pressure) has found confirmations in literature [11,12]. Thus, the TART framework remains a questionable topic in research terms.

The “variability model” (VM) is a recent empirical model of SD assessment contemplating the TART clinical signs differently as it considers the asymmetry and the restriction of movement around the joint rest position—defined as the neutral zone (NZ). This range corresponds to the initial part of movement near the rest position, and it is distinguished by the low presence of passive brakes, therefore with minimal resistance [13,14]. Stability in this sector is mostly guaranteed by the active neuromuscular action, able to control the joint movement. NZ is distinguished from the “elastic zone”, this representing the final part of the movement, which is characterized by the presence of its own constraints that maintain the mobility of the various elements within physiological limits. As the stability of the elastic zone is maintained by the passive connecting elements (e.g., capsules and ligaments), the NZ represents the biomechanical and neurological component of joint movement where the present self-regulating mechanisms can provide the possibility of interpreting the altered somatic function [7].

This type of examination appears to be more influenced by qualitative parameters rather than quantitative [15], being in line with real osteopathic clinical practice. Moreover, the VM seems to be a more reliable procedure: first of all, the assessment confined in the initial resistance-free movement could enhance tactile perception [16]; second, the gentle forces applied in the NZ reduce the possibility of a tissue deformation [17]. Despite these encouraging factors and the preliminary application of the VM validity in a clinical context [18,19], to date, there is an overall lack of evidence on this topic.

To move from a theoretical model to an explanatory theory, the reliability and the validity of the VM testing procedure have to be proven, and a comparison with gait analysis parameters can represent a proper first attempt to study its clinimetrics. The gait analysis (GA) is an easily applicable measurement tool widely used as a means of diagnosis, establishing prognosis, and planning and evaluating either a rehabilitation or treatment plan [20,21,22,23]. A recent systematic review [24] has shown that studies mostly analyze spatial–temporal parameters—especially walking velocity, cadence, and stride length—which appear to be the most relevant biomechanical parameters. Gait spatial–temporal parameters are the frequently used parameters of GA and are the most significant clinical parameters in the GA [25]. The three-dimensional instrumented gait analysis (3D-GA) is generally performed in a movement analysis laboratory and generally combines optical motion capture, force platforms, electromyography, and video recording. It provides accurate, quantitative and objective information useful to identify gait and postural problems, load asymmetries and neuromuscular abnormalities, which could not be measurable with standard clinical examinations [26].

Therefore, the aim of this preliminary study was to assess inter-examiner reliability and face, content and concurrent validity of the VM testing procedure to detect SD, through a correlation with gait analysis parameters.

## 2. Materials and Methods

### 2.1. Study Design and Methods

Three blind examiners tested 27 healthy subjects (42.5 ± 10.4) for SD on six body regions with a VM examination protocol. For this cross-sectional study, the participants were recruited from among the sanitary personnel of the Division of Neurology and Neurorehabilitation. The participants were instructed in detail about the research which was carried out in accordance with the ethical standards of the institute, following the 1964 Declaration of Helsinki and its latest amendments. Written informed consent was obtained from the participants.

Subjects were considered eligible when the following inclusion criteria were met: aged older than 18 and younger than 60; clinically negative for orthopedic or neurological diseases at the time of recruitment and without any chronic or acute disease in progress; able to walk without any assistance. Subjects were excluded if they had chronic spinal disorders and other conditions that could affect their walking ability (e.g., leg length discrepancy >2 cm; respiratory diseases or lower limb deformities).

At first, the subjects underwent a GA test, followed by a palpatory SD assessment performed with an “ad hoc protocol” referable to the VM. Three different examiners implemented the SD palpatory assessment through a blinded procedure. The raters had similar palpatory assessment experience (>10 years), and a training phase of thirty hours was performed before starting the study. Specific steps were followed during the training period to have an agreement and improve reproducibility between observers: selection of the manual assessment procedures, mutual agreement about performance, hypothesis, the final judgment and blinding of procedures. Based on the results of the above-mentioned steps, an evaluation form was developed and proven in a pilot study on 20 subjects before being used in the study.

The SD examination was performed in six different body regions, following a standardized testing procedure, assessing the quality of motion in the transverse plane. Please see the supplementary file for details (Appendix A).

### 2.2. The 3D-Gait Analysis (3D-GA)

**Figure 1:**
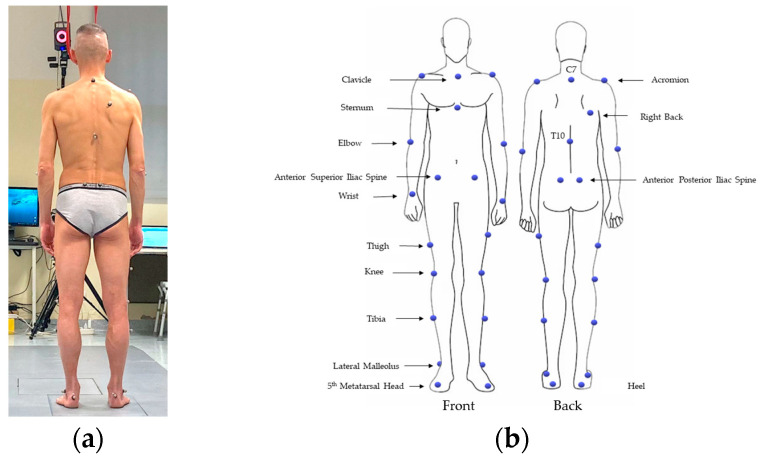
(**a**) Marker placement. (**b**) Subject equipped with the marker set.

The participants were evaluated by means of a gait analysis test at the Clinical Research Laboratory of Neurobiology and Neuropathology. Data were acquired using the gold standard technology of motion analysis for the evaluation of kinematics, consisting of an optoelectronic system with six cameras (VICON, Oxford Metrics Ltd., Oxford, UK; sample rate: 50 Hz) and two force platforms (Kistler, Winterthur, Switzerland). After collecting the anthropometric measures, the operator placed the passive markers on the subject’s skin at some specific points of reference according to the Plu In Gait model (Vicon Motion Systems) as described by Gutierrez et al. [27] and shown in Figure 1. The subject’s preparation was performed by the same operator, to limit the inter-individual errors and to ensure consistency.

The reference systems for each segment of the lower limbs were calculated starting from the 3D coordinates of the markers positioned on the pelvis, thigh, leg, and foot. Flexion–extension, abd/adduction and intra–extra rotation angles of the joints of the lower limbs were computed. The kinematic (angles) and kinetic (moments and powers) data from the 3D-GA system were not used for this study, even if available. The raw trajectories of the 3D markers were then processed using a dedicated software (Polygon Application, version 2.4, VICON, Oxford Metrics Ltd., Oxford, UK). Before starting measurements, the 3D-GA system was calibrated to ensure the accuracy of the system and to allow the estimation of the 3D marker coordinates. The average measurement error was computed on the difference between the estimated and actual distances of two passive markers fixed on the extremities of a rigid bar (actual distance: 600 mm): the calibration procedure ended with an average error within 0.3 mm (standard deviation: 0.2 mm). In this condition, the calibrated working volume was 5 m in length (*x*-axis of the laboratory reference system), 2 m in height (*y*-axis of the laboratory reference system), and 2 m along the *z*-axis of the laboratory reference system.

The considered parameters were the following:

- Cadence: number of steps in a time unit (steps/min);
- Foot off: duration of the stance phase (as a percentage of the gait cycle);
- Step length: longitudinal distance from one foot strike to the next (m);
- Walking speed: mean velocity of progression (m/s);
- Stride length: the distance between two successive placements of the same foot (m).

All the parameters were computed for the right and left lower limbs.

### 2.3. Statistical Analysis

Statistical analysis was performed with R software. Sensitivity, specificity, positive predictive value (PPV), and negative predictive value (NPV) were calculated to assess the predictive quality of NZ palpatory assessment and normal gait parameters. Fleiss’s kappa was used to assess the inter-rater reliability for SD assessment through VM testing procedure. Two models were used to assess the VM reliability. The first had 6 degrees of freedom based on the “bind” side of NZ and the three raters’ response. The second one had 7 degrees of freedom based on the severity of SD with a scale ranging from 0 to 3 (four categories) examined by three raters. Data distribution was assessed with a Shapiro–Wilk test and Kurtosis’s index. As a measure of concurrent validity, Spearman’s correlation test was used to assess the correlation between the presence/absence of SD (detected through VM testing procedure) and the gait analysis parameters. The presence of a quantitative asymmetry in movement, detected by the examiners within NZ, indicates the presence of SD and thus a positive VM test. Severity of SD was assessed with SD grading, which is reported in Appendix A.4.

To compare the patient’s general SD profile and the gait analysis parameters, the global SD score (GSD) was implemented. GSD resumes the SD grades of the tested region in a unified score, and it was calculated as the mean score of all the SD grades assessed regionally.

Univariate linear models (ULM) and multivariate linear models (MLM) were used to test the hypothesis of linear progression between GSD score and step/stride length and walking speed. For simplicity of description, R^2^ and regression coefficient have been reported along the scatter plots with model *p*-values. The *p*-values of the variables were described with appropriate symbols. Gait speed stratification for MLM was performed considering a 1.50 m/s cut-off that is slightly higher than the normal values for the study population (1.30–1.45 m/s) [28].

## 3. Results

Twenty-seven subjects fully met the inclusion criteria and participated in the experimental protocol. The participants’ characteristics were consistent with the young, healthy population. The main characteristics of gait analysis in subjects were summarized in Table 1. SD severity was tested in the cranio-cervical region (mean SD grade 1.92 ± 0.49 pt.), cervico-dorsal region (mean SD grade 1.69 ± 0.48 pt.), dorso-lumbar region (mean SD grade 1.92 ± 0.64 pt.), lumbo-pelvic region (mean SD grade 1.53 ± 0.77 pt.), right lower limb (mean SD grade 1.23 ± 0.77 pt.), and left lower limb (mean SD grade 1.61 ± 0.65 pt.). A proportion of 10/27 (37%) of patients presented normal variability within NZ in their right lower limb and 13/27 (48%) in their left lower limb.

### 3.1. Reliability

Inter-rater reliability was tested within three examiners. Table 2 and Table 3 summarize the results from NZ assessment and SD grade, respectively.

In the first assessment, the cranio-cervical region had the highest percentage of agreement (85.2%) and free-marginal k (0.78; 95% CI 0.62–0.94), whereas the left lower limb had the lowest percentage of agreement (69.1%) and free-marginal k (0.54; 95% CI 0.33–0.74). In the second assessment, the highest percentage of agreement (72.8%) and free-marginal k (0.64; 95% CI 0.47–0.81) were registered in the dorso-lumbar region. The cranio-cervical region scored the lower percentage of agreement (60.4%) and free-marginal k (0.47; 95% CI 0.29–0.66).

### 3.2. Face Validiy

All the included subjects reported their belief that the procedure they underwent was oriented to detect any possible issue related to their musculoskeletal system (altered posture, muscular tone, and motion abnormalities). The authors of the current study participated in the conceptualization and in the development of the VM testing procedure. Thus, they had considered it to be completely adapted to detect the presence of an altered SD in the body. For these reasons, face validity was reached.

### 3.3. Content Valifity

The VM testing procedure underwent the judgment of an expert panel composed of six expert osteopaths with more than 10 years of experience each in clinical practice and research. This committee convened five times, one hour each. At the end of this process, all the experts declared the VM testing procedure as a plausible strategy for detecting SD in osteopathic clinical practice.

### 3.4. Predictive Quality

Predictive quality of the VM was tested in both lower limbs. The authors summarized the result of SD testing compared with the step length normality parameters in the confusion matrix. On 54 assessments, there were 15 false negative tests and 7 false positive tests. The prevalence of abnormal step length was 0.72, and the accuracy of test was 0.59. The sensitivity and specificity were 0.62 and 0.53, respectively; the positive predicted value was 0.77, and the negative predicted value was 0.34.

### 3.5. Concurrent Validity

**Figure 2:**
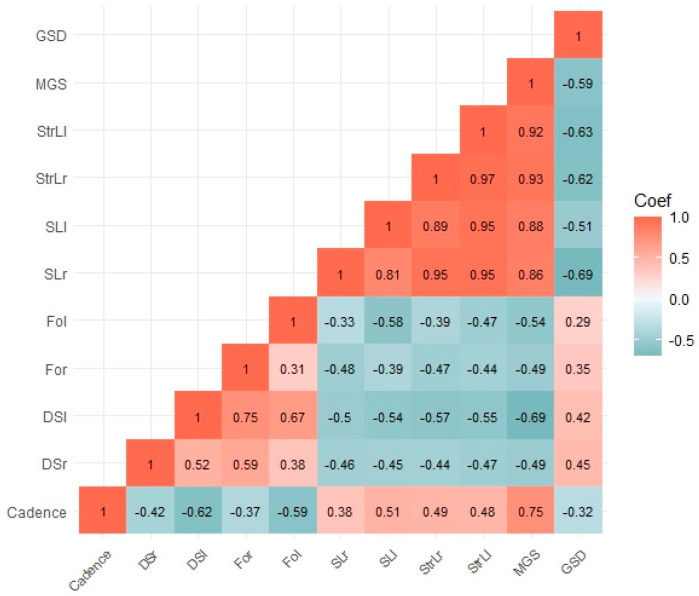
Correlation plot with heat map correlation intensity and Spearman’s correlation coefficient report. Legend: global somatic dysfunction (GSD); mean gait speed (MGS); step length right (SLr), step length left (SLl); stride length left (StrLl); stride length right (StrLr); step width left (SWl); step width right (SWr); foot off left (FOl); foot off right (FOr); double support left (DSl); double support right (DSr).

Subjects presented a mean GSD of 1.41 ± 0.38. As shown in Figure 2, the highest correlation with GSD was detected in the comparison with step length (r = −0.69 right step and r = −0.51 left step). Additionally, stride length correlated well with GSD (r = −0.62 for right step and r = −0.63 for left step). Moreover, even double support time and foot off time showed a moderate positive correlation with GSD, see Figure 2.

**Figure 3:**
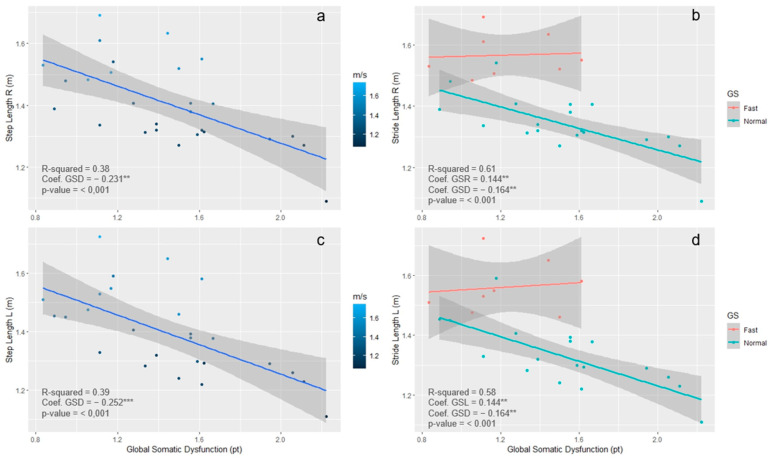
Scatter plot and prediction line plot with 95% CI boundaries. Panels (**a**,**c**) depict the ULM with scatter plots represented in different blue shades accordingly to step walking speed expressed in m/s. Panels (**b**,**d**) describe the MLM with prediction lines stratified by gait speed that was determined by the GMS cut-off at 1.5 m/s. Legend: global somatic dysfunction (GSD); mean gait speed (MGS); stride length left (StrLl); stride length right (StrLr); gait speed (GS). *p*-value 0.01 < *p* < 0.001 = **; *p* < 0.001 = ***.

As depicted in Figure 3, ULM rejected the null hypothesis of non-linear progression of GSD with stride length. Moreover, MLM showed that subjects with faster MGS (MGS > 1.5 m/s) presented a positive linear progression between the dependent and independent variables, whereas the resulting linear trend of the slower walker was stronger than that in ULM.

## 4. Discussion

Considering how clinical observation influences the entire diagnostic and therapeutic processes, SD and its clinical signs remain a distinctive concept to be clarified for osteopathic practice. So far, the above-mentioned issues in terms of reliability and validity of the osteopathic assessment procedure invited clinicians and researchers to overcome the TART framework through innovative proposals [7,8]. In this context, this preliminary study investigated the metric properties of a new SD palpatory assessment in osteopathy.

The obtained results showed how the VM presents adequate levels of both reliability and validity to detect SD. As “K” values constantly remain above the score of 0.6 for all the investigated anatomical areas—except for the left lower limb, which lies on 0.54, a value probably related to the sensitivity of the right hand—it is possible to consider VM as a substantial reliable process to detect asymmetry of movement in NZ. In other words, the VM testing procedure could be intended as a reliable method to detect motion around rest position, in the so-called NZ, and therefore useful for a somatic functional assessment. Notwithstanding this, based on the dispersion observed in k values of the examined regions, future studies should be designed to investigate the decision processes involved in the assessment of SD with VM in order to break down the entire process. A better understanding of the complex neuropsychological process of palpation—which involves executive functions, such as attention, multi-sensorial encoding, memory, and motor imagery [29]—possibly further improves the VM’s reliability, particularly when compared to the study of Degenhardt et al. [30] on the reliability of SD clinical signs palpation, which showed K values lower than 0.2 for motion asymmetry despite improving with consensus training. The authors cannot state the same in terms of “grading the severity” of the dysfunction since the K values are between 0.47 and 0.64, highlighting how the model is based on the key role of motion and its symmetry; thus, the inter-operator agreement can be classified as “moderate”. Further research should be conducted to find out new potential strategies for grading SD severity. As reported in the results section, the VM framework reached satisfactory results in terms of face and content validity. Regarding predictive validity, the findings suggest that the VM is a sensitive and positive predictive test for detection of gait step length normality ranges. This means that it is likely that there is a step length alteration when a subject presents positivity at SD examination, which is useful in planning clinical reasoning and osteopathic manipulative treatment. Conversely, there is little confidence of VM test, to detect the subject with normal step length, showing how in presence of a normal walk the palpatory evaluation does not detect SD.

Furthermore, this assessment procedure appeared to be also valid as a concurrent strategy to gait analysis considering the “good” correlations between the GSD, step length, stride length, and step walking speed. Moreover, a linear trend emerged from ULM and MLM. Particularly, the latter models evidenced some different linear trends in the fast walkers compared to normal speed walkers. Specifically, the subjects that had a faster gait speed did not present a clear linear trend between GSD and stride length. Instead, the normal GS group showed a stronger linear relationship between GSD and stride length. This fascinating perspective should be further studied, in order to implement targeted analysis at this level and on patients. Furthermore, the VM concept is coherent with this pattern that may be interpreted as a coping strategy to manage the intrinsic adaptation during walking. Such findings can be a preliminary demonstration that VM framework is a valid strategy for SD assessment, which is moderately correlated with abnormalities in terms of step and stride length. Notwithstanding this, the linear relationship between GSD and stride length cannot be generalized to the dependency of stride length to GSD due to the study level of this investigation. The results overlap with those of a recent study, in which Hill et al. [31] suggest that osteopathic structural evaluation using the Zink method, and osteopathic manipulative treatment may improve gait symmetry, reflecting functional impacts of SD. Considering other manual and manipulative therapies, spinal mobilization showed a tendency towards gait symmetry after treatment in chronic low back pain patients [32], whereas Ward et al. [33,34] measured any impact of bilateral SI joint manipulation on gait variability in asymptomatic subjects. The lack of movement variability assessed through palpation and its correlation with step and stride length showed how trunk and limb SD could influence some gait characteristics. Typical degenerative processes in the elderly highlight similar situations—e.g., older adults walk with less trunk and pelvic rotation ROM [35]—and reductions in step and stride length [36], all related to a more cautious gait. Shishov et al. [37] demonstrated that older adults with fall histories are unable to properly increase pelvis and trunk ROM when challenged to walk faster, and Kyrdalen et al. [38] have given evidence of the associations between fall risk factors and low gait speed in this regard. More rigid, less flexible pelvis–thorax coordination in the transverse plane during walking also characterizes the lower-back-pain patients [39].

The palpatory evaluation was related to the evaluation via 3D-GA—the gold standard for the movement analysis—which obtains crucial information that could not be measured with standard clinical examinations, such as the level of functional limitation due to physiology and pathology, as well as its follow-up evaluation over time [19,40,41]. Furthermore, it can help evaluate rehabilitation aimed at reducing the functional limitation due to pathology [42,43,44]. Hence, the results obtained can represent a fundamental step on the role of SD in osteopathy.

### Considerations and Implications

SD can be a characterizing clinical phenomenon revealing the intermingling of body systems and strictly bound with the musculoskeletal system, which becomes both the manifesting area and cause of the SD through the postural, traumatic, degenerative, and change aspects determined by the patient’s clinical history [2]. In this sense, the movement variability of the musculoskeletal system can detect the effect of SD, highlighting the allostatic load/overload and adaptive capacities [7]. The validity hypothesis of the VM is supported by the growing evidence concerning the fascial system, closely related to the musculoskeletal system [45,46,47,48]. The fascial system is a three-dimensional continuum of connective tissues that surrounds, permeates, and interpenetrates organs, muscles, bones, nerves, and vessels, giving the body a functional structure and allowing all the body systems to function together as a unit [45]. The alteration of the “basic regulatory system” created by the connective tissue “network” [49] could be identified in the fascial system expressing SD. Even in the fascial system, a recent study advanced a new concept: compared to the previous evidence, the interstitial space has a “different anatomy”, which justifies the free circulation of extracellular fluids [50]. Precisely for these reasons, the authors used a structural screening examination to assess distortions of the whole-body fascial system that was quantitatively assessed by GSD use. To verify the correlation with the proposed osteopathic functional palpatory assessment, gait analysis was taken into account because—as proven in different clinical contexts—the movement analysis shows different relationships between gait and trunk/pelvis function, particularly in the transverse plane [35,36,37,38,39].

The study shows how through a manual assessment osteopaths can obtain information of clinical utility, diagnostics, communication among professionals and a new role in teaching. However, it is important to also note the limits of this study: our sample size is small, preventing generalizability and predictive validity. Hence, further studies are necessary to assess VM testing procedure in larger sample size and in specific health conditions.

## 5. Conclusions

The VM testing procedure seems to be a reliable framework to detect SD in healthy people. From our results, this procedure reached satisfactory levels of concurrent validity in detecting abnormalities of gait pattern, if compared to gait analysis. Finally, the VM testing procedure has good levels of predictive validity to detect abnormalities in step length. The VM, revisiting the concept of TART, in which the primary role provides asymmetry of motion, is related to parameters strictly functional to the gait analysis and therefore to the quality of life, autonomy and the risks of falling of the subject, finding a relationship between VM, SD, and health status.

## Figures and Tables

**Table 1:** Sample characteristics and spatial–temporal parameters’ values.

Variable Name	Mean	Sta.Dev.	Min–Max
Age—years	42.5	10.4	27–55
Height—cm	169	6.53	157–180
Weight—Kg	65.9	9.90	49–85
BMI—Kg/m^2^	22.8	4.03	17.7–32.4
Stride length (right)—m	1.40	0.13	1.09–1.69
Stride length (left)—m	1.39	0.14	1.11–1.59
Step length (right)—m	0.71	0.07	0.58–0.84
Step length (left)—m	0.69	0.08	0.53–0.75
Mean gait speed—m/s	1.37	0.20	1.09–1.61
Cadence mean—steps/min	117	10.4	103–133
Double support (right)—s	0.19	0.06	0.09–0.36
Double support (left)—s	0.19	0.05	0.10–0.29
Foot off (right)—% of stance	59.6	2.59	54.4–64.2
Foot off (left)—% of stance	60.2	1.98	56.7–64.7

**Table 2:** Percentage of agreement and free-marginal k with 95% CI of the NZ.

Variable	Overall % of Agreement	Fleiss’s k	95% CI
Cranio-cervical region	85.2	0.78	0.62–0.94
Cervico-dorsal region	75.3	0.63	0.44–0.82
Dorso-lumbar region	79.0	0.69	0.50–0.87
Lumbo-pelvic region	75.3	0.63	0.44–0.82
Right lower limb	79.1	0.69	0.50–0.87
Left lower limb	69.1	0.54	0.33–0.74

**Table 3:** Percentage of agreement and free-marginal k with 95% CI of the SD grade performed by three raters.

Variable	Overall % of Agreement	Fleiss’s k	95% CI
Cranio-cervical region	62.9	0.51	0.34–0.68
Cervico-dorsal region	60.4	0.47	0.29–0.66
Dorso-lumbar region	72.8	0.64	0.47–0.81
Lumbo-pelvic region	62.9	0.51	0.34–0.68
Right lower limb	66.7	0.56	0.38–0.74
Left lower limb	70.3	0.60	0.42–0.79

**Figure A1:**
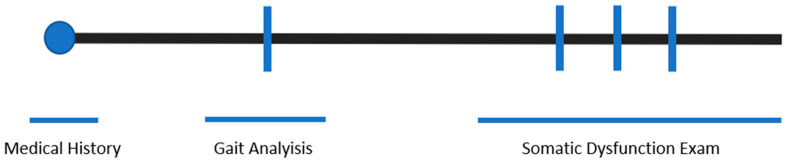
Patients’ examination procedure.

**Figure A2:**
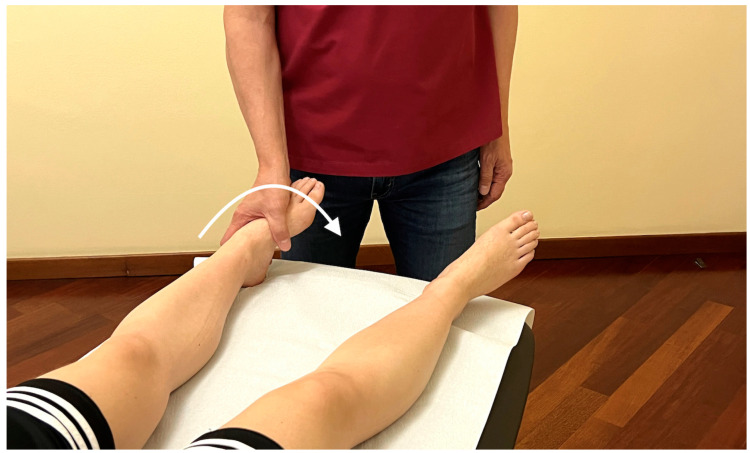
Lower limb: rotatory test of the hip and torsion of the lower limb.

**Figure A3:**
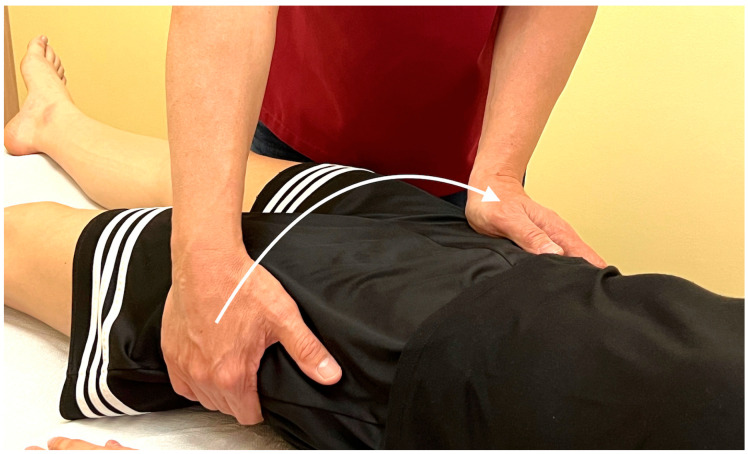
Lumbo-pelvic region: rotation assessment.

**Figure A4:**
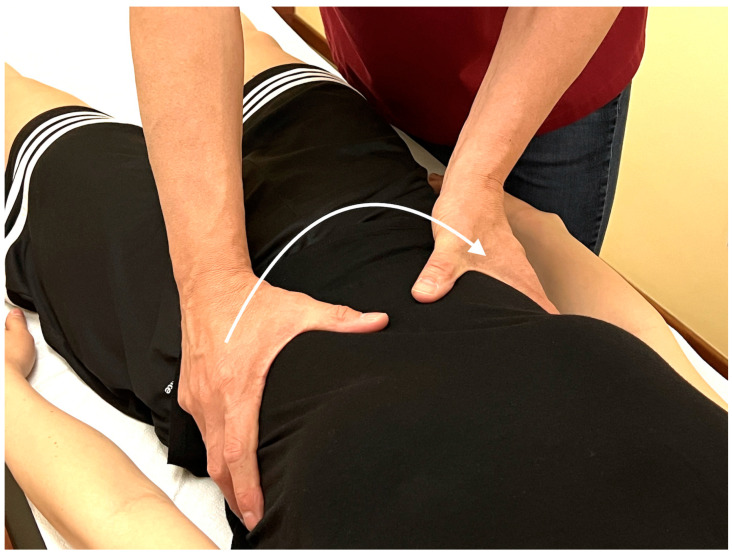
Lower dorso-lumbar and thorax region: rotation assessment.

**Figure A5:**
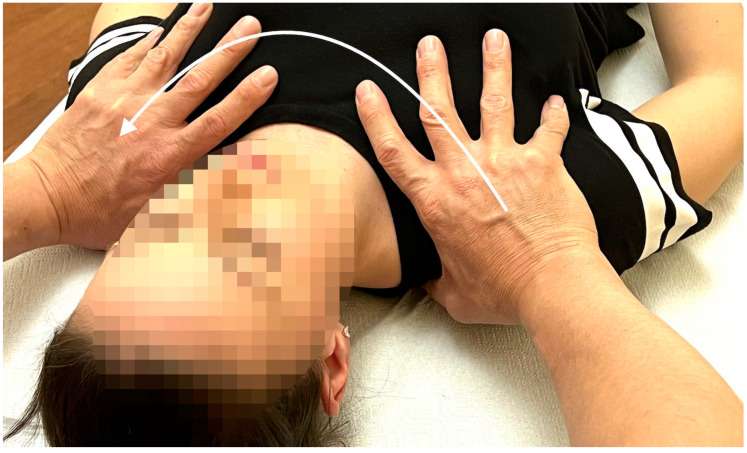
Upper thorax and cervico-dorsal region: rotation assessment.

**Figure A6:**
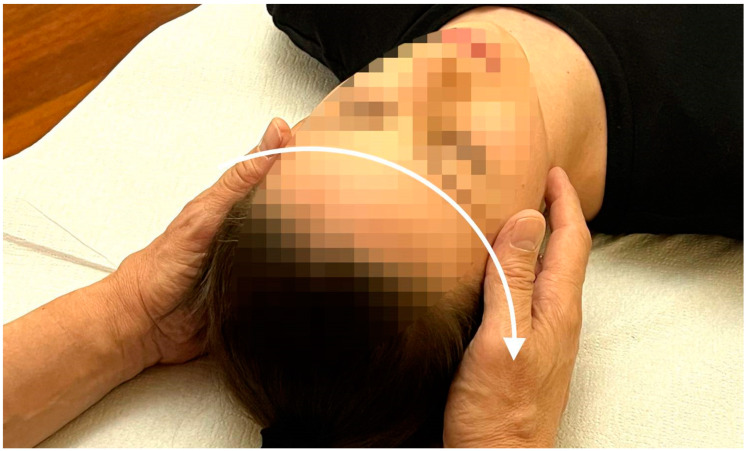
Cranio-cervical region: rotation assessment.

## Data Availability

The datasets generated during and/or analyzed during the current study are available in the Zenodo repository with the identifier. https://doi.org/10.5281/zenodo.10207716, accessed on 27 November 2023.

## Appendix A

### Appendix A.1. Procedure of Investigation

Here is the experimental protocol that patient underwent during the investigation. The experimental team was composed of three osteopathic practitioners with more than 10 years of experience each and one bioengineer. During the examination each patient followed the procedure as depicted in the Figure A1.

### Appendix A.2. Somatic Dysfunction Definition

Impaired or altered function of related components of the body framework system: skeletal, arthrodial, and myofascial structures and their related vascular, lymphatic, and neural elements. It is characterized by positional asymmetry, restricted range of motion, tissue texture abnormalities, and/or tenderness. The positional and motion aspects of somatic dysfunction are generally described by: (1) the position of a body part as determined by palpation and referenced to its defined adjacent structure, (2) the directions in which motion is freer, and (3) the directions in which motion is restricted. Somatic dysfunction is treatable using osteopathic manipulative treatment [4].

### Appendix A.3. Variability Model Definition

The variability model (VM) is a new theoretical model created for somatic dysfunction (SD) assessment. At the base of VM, there is the analysis of the joint motion variability in the range around the rest or neutral position in the so-called neutral zone (NZ). Specific palpatory technique allow to assess if the movement within NZ is symmetric in joint play. In case of evident poor symmetry of test response, SD is palpatory diagnosed. Vice versa, the presence of the symmetric movement variability expresses normal function.

### Appendix A.4. Somatic Dysfunction Classification According to VM

Regional tissue texture and tenderness assessment composes the classification of SD. Altered tissue texture is defined as reduced elastic deformation of soft tissue tested with compression (linear increment of resistance and not exponential/logarithmic increment) or traction (reduced or no-latency between traction extremities and not latency).

- Grade 0—No SD: symmetric variability of motion in the NZ present (symmetric motion);
- Grade 1—Mild SD: asymmetric variability of mobility in the NZ (asymmetric motion);
- Grade 2—Moderate SD: asymmetric variability of motion in the NZ + altered tissue texture or tenderness;
- Grade 3—Severe SD: asymmetric variability of motion in the NZ + altered tissue texture + tenderness.

### Appendix A.5. Palpatory Procedure

The palpatory assessment of SD was performed as depicted in the following Figure A2, Figure A3, Figure A4, Figure A5 and Figure A6:
